# Use of a leaf chlorophyll content index to improve the prediction of above-ground biomass and productivity

**DOI:** 10.7717/peerj.6240

**Published:** 2019-01-11

**Authors:** Chuang Liu, Yi Liu, Yanhong Lu, Yulin Liao, Jun Nie, Xiaoliang Yuan, Fang Chen

**Affiliations:** 1 Key Laboratory of Aquatic Botany and Watershed Ecology, Wuhan Botanical Garden Chinese Academy of Sciences, Wuhan, Hubei, China; 2 University of Chinese Academy of Sciences, Beijing, China; 3 Soil and Fertilizer Institute, Hunan Academy of Agricultural Sciences, Changsha, Hunan, China; 4 China Program of International Plant Nutrition Institute, Wuhan, Hubei, China

**Keywords:** Rice, Leaf chlorophyll content index, Rice biomass simulation

## Abstract

Improving the accuracy of predicting plant productivity is a key element in planning nutrient management strategies to ensure a balance between nutrient supply and demand under climate change. A calculation based on intercepted photosynthetically active radiation is an effective and relatively reliable way to determine the climate impact on a crop above-ground biomass (AGB). This research shows that using variations in a chlorophyll content index (CCI) in a mathematical function could effectively obtain good statistical diagnostic results between simulated and observed crop biomass. In this study, the leaf CCI, which is used as a biochemical photosynthetic component and calibration parameter, increased simulation accuracy across the growing stages during 2016–2017. This calculation improves the accuracy of prediction and modelling of crops under specific agroecosystems, and it may also improve projections of AGB for a variety of other crops.

## Introduction

Above-ground biomass (AGB) is important for predicting plant growth and development within different ecosystems. It is also used to evaluate the productivity of many crops. Biomass dynamics are generally controlled by abiotic and biotic factors such as solar radiation, temperature, rainfall, soil nutrient, leaf area index (LAI), vegetative type, and human activities (Boutton, Tieszen & Imbamba, 1988; Keeler et al., 2015; Jomura et al., 2015; Van Der Sande et al., 2017). Crop AGB is affected by genetics, physiology, and environmental factors (Mavromatis et al., 2002), and it is usually regarded as an index for estimating yield and economic benefits in agricultural ecosystems. It is well known that the leaf blade is one of the most important parts of a plant. Leaf blade helps supply remobilized nitrogen in the development of green plant biomass and it affects photosynthesis by transforming solar radiation to chemical energy (Abbad et al., 2004; Bailey & Leegood, 2016; Bianculli et al., 2016).

Crop simulation models are an indispensable tool in agricultural research and policy analysis, and they can be used to predict crop growth and AGB (Jing et al., 2007). Moreover, they are used for the development of decision support system for management, estimation of crop yields and simulations of plant growth in agriculture (Xiong et al., 2008; Adhikari et al., 2017). Moriondo, Maselli & Bindi (2007) summarized the calculation of AGB based on photosynthetically active radiation (PAR_*i*_) intercepted by the canopy leaves. However, the current AGB model only consideres PAR_*i*_, LAI, and solar radiation. In fact, AGB is affected by many biotic or abiotic factors. Temesgen et al. (2015) reported that the accuracy of forest biomass models were determined by the selection of sample plots and sample trees when developing or calibrating models. Landsberg & Sands (2011) focused on factors that influenced photosynthesis and the accompanying process of carbon assimilation into ecosystems. Leaf development and senescence, and associated leaf demography (i.e., the distribution of leaf ages), can cause seasonal shifts in both leaf quantity (i.e., canopy leaf area) and leaf quality (i.e., per-area photosynthetic capacity) (Doughty & Goulden, 2008; Wu et al., 2016).

Leaf chlorophyll is a key indicator of leaf greenness, and it is often used to investigate leaf nutrient deficiencies and changes in chlorophyll (Ali et al., 2017). Canopy chlorophyll content is also an indicator of seasonal carbon uptake in forest ecosystems (Croft et al., 2015). Significant correlations between chlorophyll content and leaf nitrogen have been reported in many agricultural crops (Wang et al., 2014; Kalacska, Lalonde & Moore, 2015). Chlorophyll content, LAI and leaf dry weight are positively influenced by fertilizer application, especially nitrogen (Hokmalipour & Darbandi, 2011). Dordas & Sioulas (2008) reported that after fertilization, the low N supply would be remobilized for safflower growth, resulting in acceleration of leaf senesce and decreased chlorophyll content. Quantifying leaf pigmentation using this noninvasive optical method is fast and easy, and produces reliable estimates of relative leaf chlorophyll when compared to traditional and chemical methods (Richardson, Duigan & Berlyn, 2002). Miri (2009) reported that chlorophyll content index (CCI) was significantly and positively correlated with grain yield and a harvest index of wheat in Iran. Chlorophyll is not only used as a substitute for leaf nitrogen content, but also as an essential indicator of N-deficiency in agriculture (Cerovic et al., 2012). The relationship between CCI and chlorophyll has been found to be linear in wheat and Asian pear tree. (Ghasemi et al., 2011; Lunagaria, Patel & Pandey, 2015; Kaur et al., 2015). Furthermore, CCI for relative chlorophyll content can be used as a decision support tool for N-fertilization of crops, and even more it may be used for improving the estimation of crop yield and biomass.

The availability of estimated CCI parameters that describe crop canopy characteristics is important for the development of a methodological framework that may be operationally adopted for regional scale assessment of crop biomass. Our study aims to (1) monitor the changes in LAI, PAR_*i*_, CCI, and AGB in different growing phases, (2) predict AGB based on PAR_*i*_ by using CCI and LAI, and to analyze the relationship between observed and simulated values, and (3) improve the method for formulating AGB in early and late rice crops to increase the accuracy of simulation, based on CCI, LAI, and PAR_*i*_.

## Materials and Methods

### Site description

The Wangcheng Field Experimental Station is in Changsha in Hunan Province, China (28.37° N, 112.80° E). The field experiments were performed for four growing seasons in 2016 and 2017 using early and late rice cultivars of Xiangzaoxian 45 and Fengyuanyou 272, respectively. The soil was a typical hapli-stagnic anthrosols developed from quaternary red clay. The soil pH, organic matter, available nitrogen, available phosphorus, and available potassium were 6.6, 36.33 g kg^−1^, 118.65 mg kg^−1^, 10.19 mg kg^−1^, and 107.03 mg kg^−1^, respectively.

**Figure 1:**
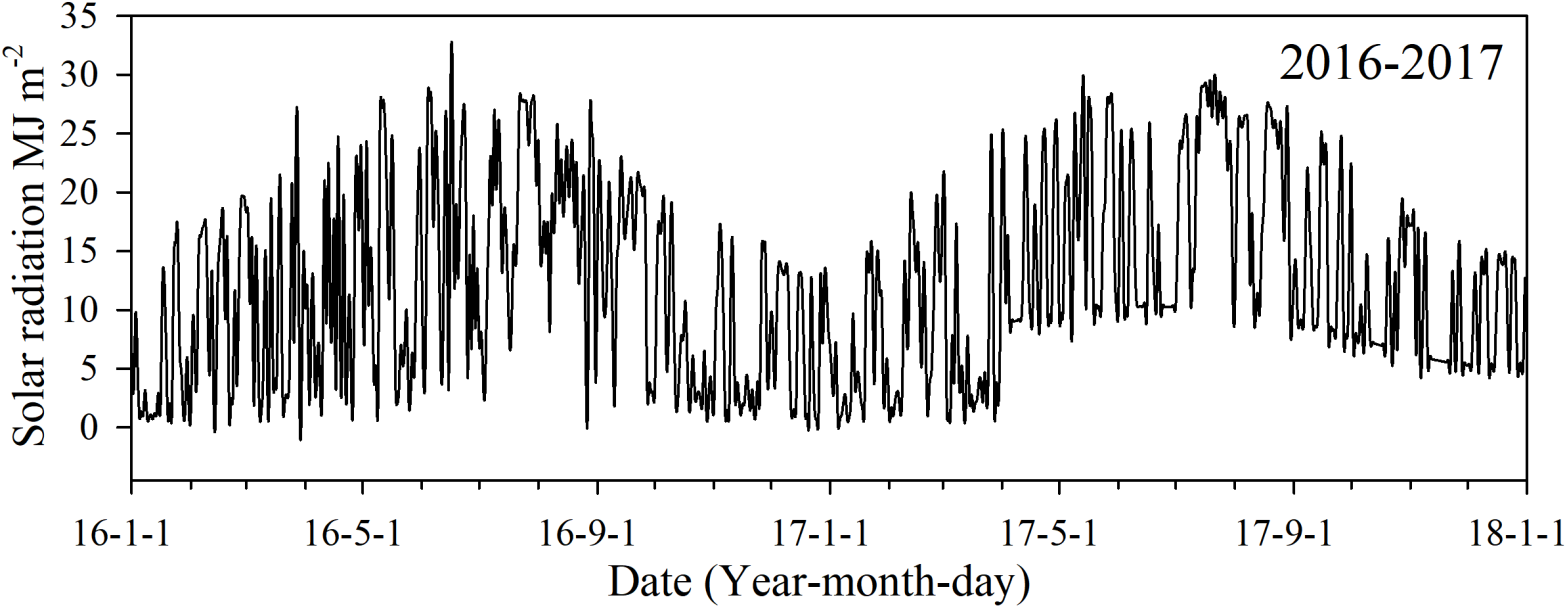
Daily solar radiation in 2016 and 2017 during the monitored growing seasons in Changsha, China.

Daily solar radiation during each of the growing seasons was obtained from China Meteorological Administration (http://www.cma.gov.cn) (Fig. 1). Daily Solar radiation in 2017 was calculated by Angstrom’s (1924) empirical formula:

$${R_s} = {R_{{\rm max}}}\left( {{a_s} + {b_s}\displaystyle{n \over N}} \right)$$

where *R_s_* was the total incoming radiation during the day, *R*_max_ was the theoretical incoming radiation on a clear day, *a_s_* and *b_s_* were empirical constants 0.25 and 0.5, respectively, as the United Nations Food and Agriculture Organization’s, *n* was the number of daily sunshine hours from the local weather station, and *N* was the greatest number of hours of sunshine.

### Experimental design and fertilization treatments

Nine fertilizer treatments arranged in a randomized complete block design were incorporated into the experimental designed: T1 (urea: zero kg ha^−1^), T2 (controlled release urea: 150 kg ha^−1^), T3 (urea: 120 kg ha^−1^), T4 (urea: 150 kg ha^−^^1^), T5 (urea: 180 kg ha^−1^), T6 (controlled release urea: 120 kg ha^−1^ and urea: 30 kg ha^−1^), T7 (T4 + straw return), T8 (T6 + straw return), and T9 (Fertilization recommended by Nutrient Expert system; NE®, Beijing, China). Each treatment was applied in a plot of 20 m^2^ (4 m × 5 m) with three replications. In application treatments for urea, 20% of the total *N* was applied as a top application after sowing, and the remaining 80% was applied as a basal dressing.

### Plant analysis

Plant samples were collected, dried at 105 °C for 30 min and then dried at 70 °C for 3–5 days until they reached a constant weight. AGB in each plot was expressed in terms of kg dry matter per ha.

### Leaf area index

The area of each fresh leaf was measured immediately after sampling, and then calculated using the threshold-based pixel count method with Image J software (Martin et al., 2013; Easlon & Bloom, 2014). LAI values of each plot were calculated by multiplying the leaf area of each plant by the plant density (225,000 plants ha^−1^) (i.e., LAI = leaf area (m^2^ plant^−1^) × 225,000 (plants ha^−1^)/10,000 (m^2^ ha^−1^)) (Yi et al., 2010).

### Chlorophyll content index values

A portable, nondestructive, and lightweight instrument (CCM-200; Opti-Sciences Inc., Hudson, NH, USA) was used to estimate CCI for early and late rice, and it provided instantaneous, *in situ* information. The CCM-200 was adopted to take CCI values from the first fully expanded functional leaf on each plant. Total of 10 plants were measured randomly in each plot, and three CCI readings per leaf, including one reading around the midpoint of leaf blade and two readings three cm apart from midpoint. These values were averaged for the mean CCI reading of each leaf (Peng et al., 1993). CCI in our research was standardized with the following equation:

(1) $$\Delta {\rm CCI} = \displaystyle{{x - {\rm min}} \over {{\rm max} - {\rm min}}}$$

where *x* was the observed CCI values, max and min were the sample maximum and minimum of CCI in each growing stage.

### Intercepted photosynthetically active radiation and PAR_*i*_ using CCI

Yang et al. (2004) reported that PAR was 50% of the total incident solar radiation (*R*), and the amount of PAR that was intercepted by the plant canopy can be computed using the following exponential function:

(2) $${\rm Model }1:{\rm PA}{{\rm R}_i} = {\rm }\Sigma \,0.5R{\rm }\left( {1{\rm }- {\rm }{e^{ - k{\rm LAI}}}} \right)$$

where *R* is the incoming total solar radiation (MJ m^−2^ d^−1^), *k* is the light extinction coefficient, which equals 0.60 for rice from Monteith (1969), and LAI (m^2^ leaf m^−2^ ground).

Here, we propose and evaluate a modification to the model of Yang et al. (2004) that accounts for the effects of variations in leaf chlorophyll content as follows:

(3) $${\rm Model }2:{\rm PA}{{\rm R}_i} - {\rm CCI } = {\rm }\Sigma \,0.5R{\rm }\left( {1{\rm }- {\rm }{e^{ - k{\rm LAI\Delta CCI}}}} \right)$$

where ΔCCI is normalized CCI as defined above.

AGB was computed by using Moriondo, Maselli & Bindi (2007):

(4) $${\rm AGB} = \sum_{i = 1}^N {{{\rm \varepsilon }_i}{\rm PA}{{\rm R}_i}{\rm Ra}{{\rm d}_i}}$$

where ε_*i*_ and Rad_*i*_ are the radiation use efficiency (RUE, g MJ^−1^) and PAR at day *i*, respectively, and *N* is the number of simulation days. In particular, RUE was kept constant at 2.9 g MJ^−1^ during all stages of growing rice (Peng et al., 2014).

### Statistical analyses for model evaluation

To evaluate the performance of Model1 and Model2, a subset of the statistical diagnostics suggested by Sutton et al. (1997) were used to compare the simulated and measured data. Here, we define the error as measured minus simulated data. Standard goodness of fit diagnostics were calculated to evaluate the models: (a) root mean squared error (RMSE, 0 (optimal) ≤ RMSE < +∞), (b) mean absolute error (MAE, optimum, 0 (optimal) ≤ MAE < +∞), (c) modelling efficiency (EF, −∞ < EF < 1 (optimal), where negative EF values indicate that the mean of the measured data is a better predictor than the model results (Sutton et al., 1997), (d) relative error and (e) correlation coefficient (*r*) between the measured and simulated data (−1 ≤ *r* ≤ 1). Although RMSE and MAE relay similar characteristics about model accuracy, MAE is more resistant to outlying errors.

## Results

### Changes in rice leaf characteristics and relationship to photosynthetic dynamics

**Figure 2:**
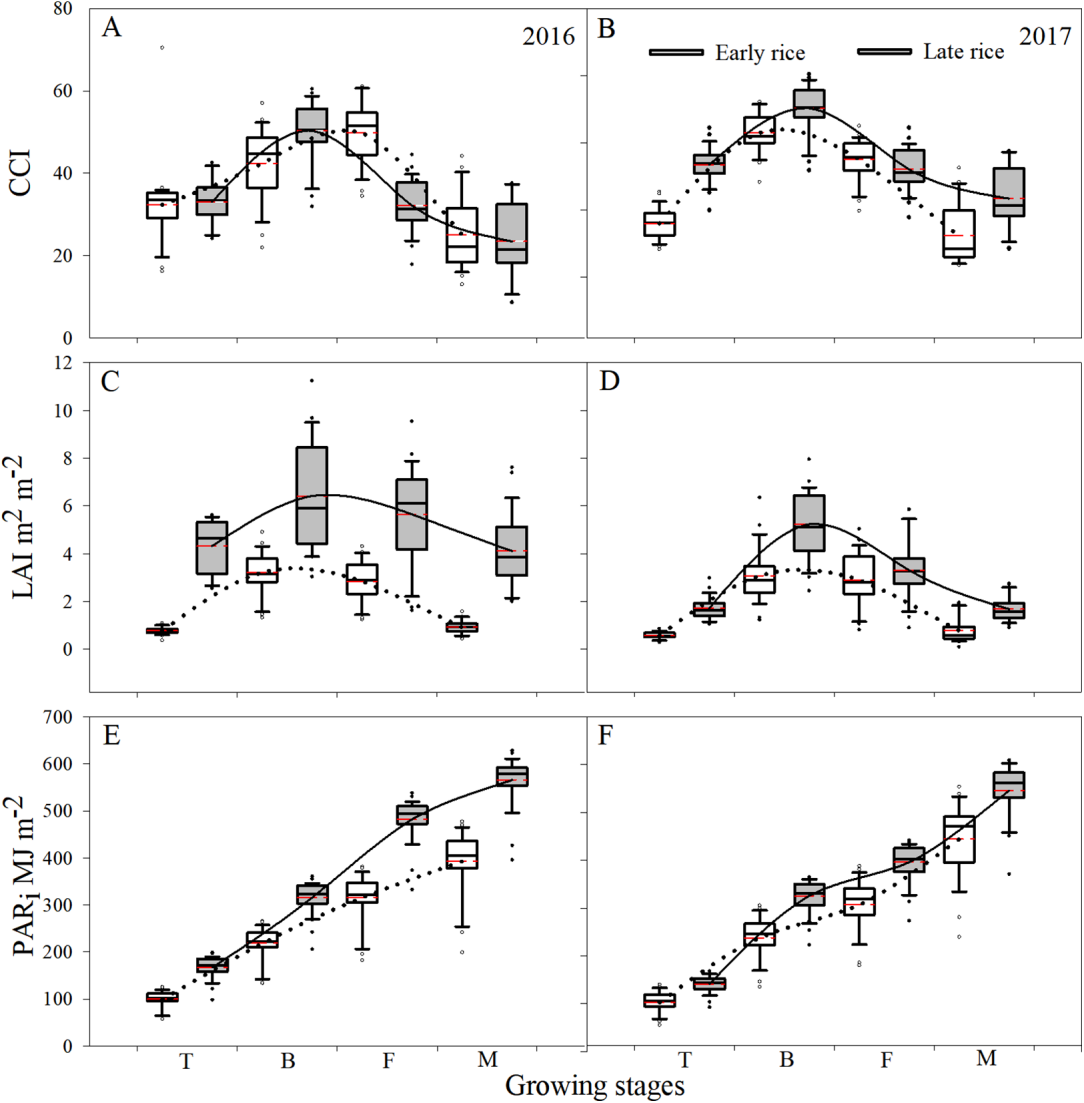
Boxplot of changes in CCI (A and B), LAI (C and D), and PAR_*i*_ (E and F) among the different growing stages of rice during 2016 and 2017 in Changsha, China. The upper and lower hinge of box indicated the 75th percentile and 25th percentile of the data set, respectively. The line in the box indicates the median value of the data, and the red line represents the mean of the data. The solid and dotted curve represented the mean value of the fitted curve across the growing stages.

Leaf CCI values at the different growing stages were sensitive to relative chlorophyll content, which increased and then decreased with the shift in phenology. During 2016–2017, across the growth stages for early rice, the median CCI values for the tillering stage, booting stage, filling stage, and maturity stage were 21.1, 43.1, 41.1, and 18.9, respectively. Across the growth stages for late rice, the median value of CCI for the booting stage reached a maximum of 46.6, however, the median values for the filling stage and the maturity stage were 31.1 and 21.4, respectively. Across the growing stages, the mean CCI value for the early rice filling stage was higher than late rice by 17.6 and 4.6 for 2016 and 2017, respectively, however, the mean values of early rice for the tillering stage, booting stage, and maturity stage were lower than for late rice (Figs. 2A and 2B).

The temporal trend in the average LAI was similar to CCI which changed with phenology. As a result, during 2016–2017, across the growing stages, the median values for late rice LAI at the tillering, booting, filling, and maturity stages were higher than those of early rice by 1.3, 2.5, 1.0, and 1.7 m^2^ m^−2^, respectively. However, the maximum LAI value for early rice was still far lower than the minimum of late rice at the tillering stage. Across the stages, the greatest LAI values were 3.2 and 5.7 m^2^ m^−2^ for early and late rice, respectively, and occurred at the booting stage. The corresponding and fitted curves for late rice was higher than for early rice across all growing stages (Figs. 2C and 2D).

Photosynthetically active radiation increased with the changes in phenology during 2016–2017, which suggested that the increase in LAI was due to enhance interception of solar radiation which further promoted the production of biomass across the growing stages. Nevertheless, PAR_*i*_ was different between early and late rice during all developing stages, and the mean PAR_*i*_ for late rice was higher than for early rice (Figs. 2E and 2F). In addition to other factors such as cultivar changes, photosynthetic ability of the leaves to intercept solar radiation contributed to the shifts in AGB during the growing stages of rice.

### Possible uncertainties of simulation obtained by AGB with and without considering CCI

There is a heritage to the model (Model1) whereas conventional or established suggests it’s what is usually used by other researchers (Yang et al., 2004). However, some versions of Model1 are field-specific and stage-specific, and therefore they are not easily applied to other fields to predict AGB. Using variations of a CCI in the improved AGB method (Model2) obtained accurate results between simulated and observed crop biomass in a paddy field. Moreover, the leaf CCI, which is used as a biochemical photosynthetic component and calibration parameter, increased the simulation accuracy across the growing stages during 2016–2017.

**Figure 3:**
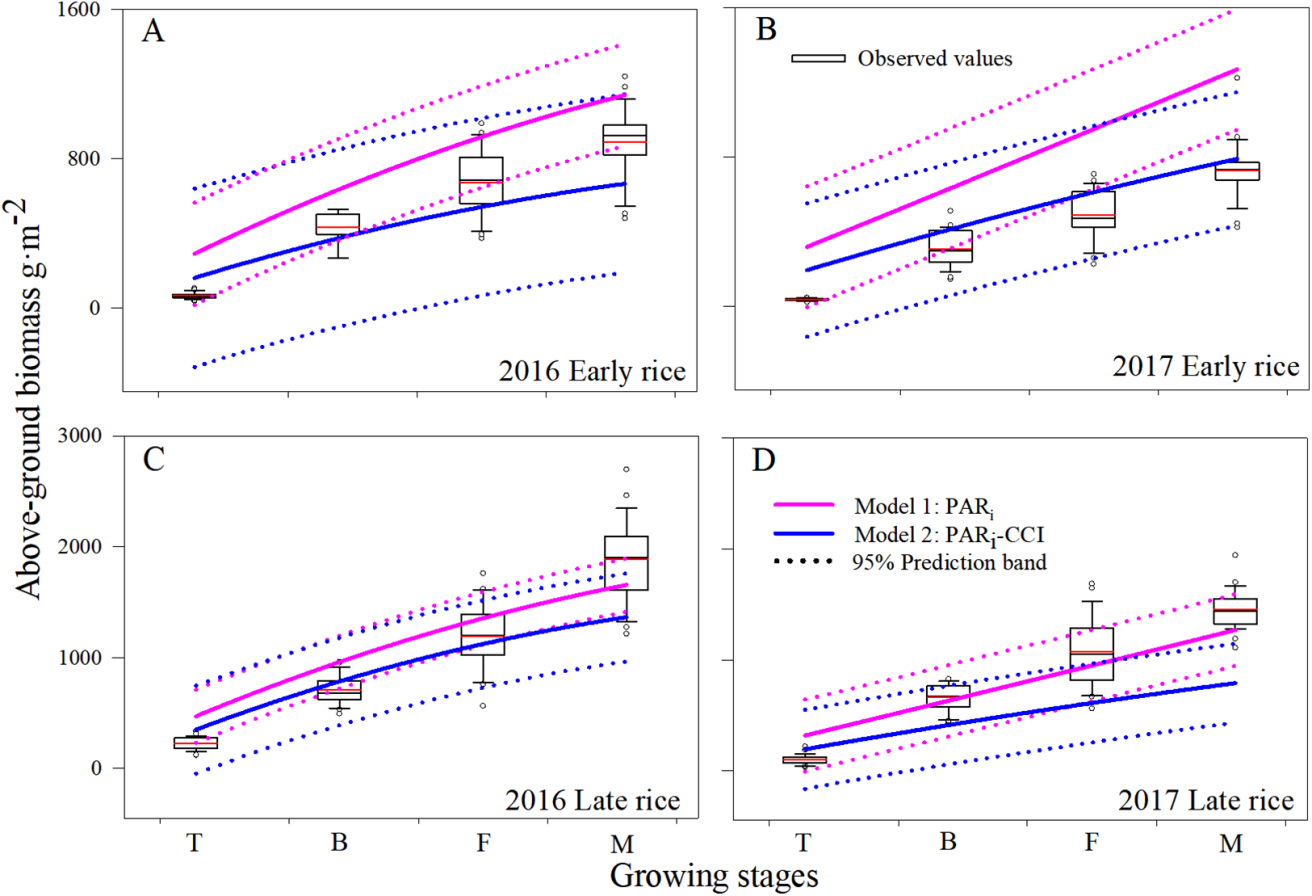
Comparison of AGB (g m^−2^) estimated from Model1 and Model2. Boxplot (A, B, C and D) of changes in AGB among the different growing stages during 2016 and 2017 in Changsha, China. The upper and lower hinge in each box indicates the 75th percentile and 25th percentile of the observed data, respectively. The dotted line is the 95% prediction band of the second order polynomial fit the model.

To assess uncertainties related to Model2, our evaluation for use with CCI, LAI, and PAR_*i*_, all of which were added as representations of photosynthesis. Across all growing stages during 2016–2017, the average accumulation of AGB increased nonlinearly with shifts in phenology, and average biomass reached a maximum when the rice reached maturity. Model1 predictions of AGB for all rice seasons, except the 2017 late rice, were poorly related to measured AGB, particularly at the tillering stages. However, Model1 performed better for late rice than for early rice (Fig. 3D). There are a few plausible explanations for Model1’s poor early rice simulation results. First, the disturbance resulting from transplanting the plants was not considered in the model though it would affect the AGB. Second, Model1 estimates were based only on canopy leaf area which may not have been parameterized and formulated exactly. Third, leaf physiology was not taken into account in Model1. Finally, the location of light interception for chlorophyll was on the leaves, where the leaf area of was over-estimated across all growing stages, which probably caused an over estimation of AGB.

The mean AGB for each of the growing stages, as predicted using Model1 and Model2, showed a similar pattern of increase with increased plant growth. Across all growing stages, predicted mean estimates of AGB were 371.1–1,413.5 g m^−2^ and 258.7–1,005.6 g m^−2^ for Model1 and Model2, respectively. In practice, the mean observed values varied from 106.4 to 1,240.3 g m^−2^. Estimates using Model1 were higher for AGB due to inclusion of only part of the leaf area; Model2 estimates using CCI as a calibration parameter greatly reduced over-estimation and the accuracy of the simulation.

### Model validation

**Figure 4:**
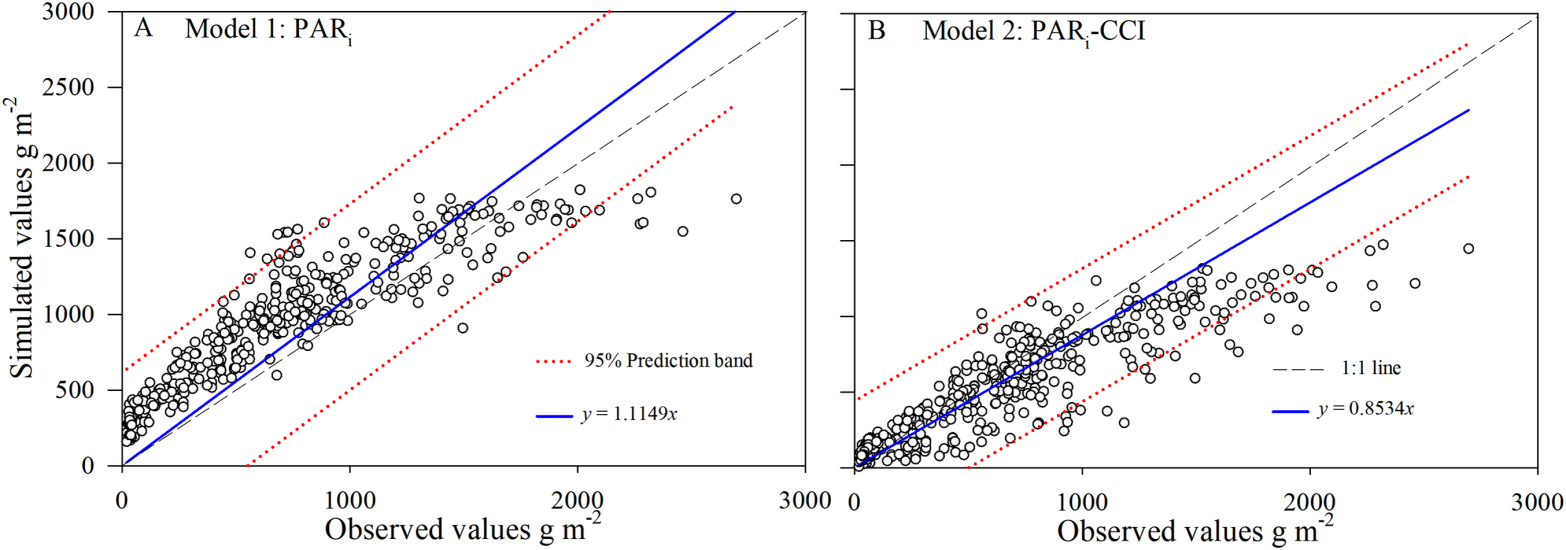
Simulated and observed values (A and B) of above-ground biomass (AGB) for rice across datasets during 2016–2017 in Changsha, China. The dotted lines were 95% prediction band.

The statistical diagnostics (Table 1) confirm the tendency for Model2 simulations to describe the measured data marginally better than Model1 simulations. The correct estimation of AGB is crucial to assess the validity of the crop simulation models. The comparison of observed and simulated results showed that Model2 using CCI simulated the changes of AGB with crop growth for all of field experiments during 2016–2017 (Fig. 4). Simulations of biomass yields less than 1,000 g m^−2^ in the Model2 were better predicted when compared to Model1. However, both models under-predict AGB to some degree at around the 1,500 g m^−2^. This underestimation of AGB is more apparent from both models over 2,000 g m^−2^.

**Table 1:** Statistical analysis of Model1 and Model2 performance on dynamics of AGB.

Criteria	Model1 (*n* = 432)	Model2 (*n* = 432)
*r*	0.88	0.91
RMSE (RMSE95%)	0.48	0.39
EF	0.62	0.75
RE (RE95%)	−135.64	−55.49
RMSE	328.51	266.97
MAE	288.76	195.91

Simulated AGB from Model1 and Model2 were compared with the observed data at the early rice and late rice seasons for the period between tillering and maturity (Fig. 3). The overall accuracy of each model’s performance is given by the statistical diagnostics (Table 1). In general, the simulations for AGB appear mostly accurate from both Model1 and Model2. Model2 predicts the total biomass well at low AGB values (<1,000 g m^−2^), while Model1 predicts biomass well during the mature stage. This behavior is more apparent for Model2 in the late rice simulation, especially with respect to the under-prediction at high biomass. The prediction band (95%) for Model2 is narrower than for Model1, and the results showed that the simulation data from Model2 is more concentrated and accurate. Overall, this implies that Model2 provides improved simulation of AGB compared to Model1, resulting in a higher sensitivity and robust identifiability of the rice filling stage.

## Discussion

### Effects of CCI, LAI, and PAR_*i*_ on AGB

Crop growth conditions are often oversimplified in crop growing models. The architecture of these models includes some uncertainties, such as crop and meteorological parameters, which result in biased crop growth and yield simulation (Huang et al., 2015). In this study, we tried to reduce this uncertainty by using CCI to calibrate the AGB model. We used CCI as a comprehensive indicator by mutating the original CCI values using a method similar to standardizing a calibrated parameter (Fig. 3).

Yamori et al. (2016) reported that leaf photosynthesis was the most significant factor for grain yield and biomass. In contrast, few studies have focused on the relationship between photosynthetic capacity of crops and biochemical photosynthetic components such as leaf chlorophyll or carotenoid content to develop a biomass or yield model (Houborg et al., 2013). Chlorophyll is an important part of the Calvin–Benson cycle, and it is responsible for harvesting light during photosynthesis, which results in the excitation of electrons that are used to drive the production of nicotinamide adenine dinucleotide phosphate and chemical energy in the form of adenosine triphosphate (Croft et al., 2017). It also provides an indication of nutritional status as a result of nitrogen fertilizer, which serves as a reliable means to estimate the function of *N* fertilizer (Martínez & Guiamet, 2004).

Leaf area index had been widely used as a biophysical variable for vegetation to estimate yields and crop growth in agricultural models, such as WOFOST (Huang et al., 2015). It also represents the ability to intercept solar radiation, which drives CO_2_ assimilation and dry matter accumulation, and it is considered an important predictor of potential grain yield (Huang et al., 2016). Our results indicated that the observed quadratic response of LAI across the growing stages during 2016–2017 was similar to the changes in CCI values. Our LAI data exhibited the same tendency to rise and then fall as the growing stages progressed, where LAI and grain yield reached peak values in the booting stage and then decreased in the mature stage. Ali et al. (2016) reported that there was an association between LAI and total grain yield, and that LAI increased with higher yield, greater tiller numbers, and taller plants in rice. Tsimba et al. (2014) suggested that planting date had a significant impact on LAI, and planting too early and too late resulted in the lowest LAI values. Previous research has shown a correlation between LAI and the normalized difference vegetation index (NDVI) or net primary productivity (NPP) (Lefsky et al., 2005; Powell et al., 2010). The increase in LAI enhanced the NDVI and NPP, which promoted the response of canopy transpiration in early and late rice.

Photosynthetically active radiation was used to estimate LAI values, and its seasonal changes varied with canopy LAI. In our analysis, its variation consistently increased and then slowly levelled off during all growing stages. Changes of PAR_*i*_ in early rice were similar to that of late rice across the growing stages. PAR_*i*_ values in late rice were higher than that of early rice because the solar radiation quality and quantity into late rice exceeded that to early rice (Fig. 1). Suitable solar radiation was conducive to the leaf interception of radiation, which promoted leaf photosynthesis and further increased AGB.

### Improvement for the prediction of AGB and productivity

Crop biomass determination is traditionally based on measured data; we used a the field harvest estimation method in our study. This method is accurate, but it is more expensive and time-consuming. Yi et al. (2010) reported that spectral estimation of green plant biomass had some advantages, but it was easily influenced by many factors such as water, light, soil, and differences in vegetation. Compared with the traditional sampling method, the remote sensing methods can quickly and accurately estimate AGB, and are able to monitor site-specific changes in ecosystems in real time. However, the spectroscopy method might be difficult for estimating AGB under a flooded area. Photosynthetic rates are related closely to leaf chlorophyll content, and the chlorophyll content is positively correlated with the CCI of the leaves (Parry, Blonquist & Bugbee, 2014). The relationship between CCI and chlorophyll was found to be linear in wheat (Lunagaria, Patel & Pandey, 2015; Kaur et al., 2015), and Ghasemi et al. (2011) also reported a linear CCI-chlorophyll relationship for Asian pear tree. Furthermore, the increase in chlorophyll can be measured easily and quickly over a large area using portable instruments such as the CCM-200 meter.

Based on the statistical diagnostics (Table 1) of Model2 which used CCI as a biological parameter was better than Model1. In field studies, leaf chlorophyll content was positively associated with the level of photosynthesis in leaves in China (Xiao et al., 2012), and CCI has been positively associated with leaf nitrogen content in rice (Atashbar et al., 2008). In our study, CCI was introduced as an important physiological parameter for improving estimation of AGB, and it performed well across the growing stages. Model2 with CCI as a parameter improved the simulation accuracy before the filling stage, and good statistical diagnostics results were obtained (Table 1). Although acquisition of model data using CCI worked well, some uncertainties remain. Model2 did not show good results in the maturity stage, especially for the late rice season in 2017, which means it is not suitable for simulation of biomass after the filling stage (Fig. 3). This may have been due to leaf senescence (Xu et al., 2017), nutrient translocation (Hou et al., 2015), and leaf fibrosis (Tang et al., 2003) after the filling stage, which led further to a rapid reduction in leaf chlorophyll content. Also, the accumulation of material in the middle and late period for rice was related closely to the stability of the chlorophyll content in functional leaves, and its instability led to a decrease in the leaf area of the rice population, which affected the production of photosynthetic products (Yang et al., 2001). Therefore, our results showed that Model2 decreased the difference between simulated and observed biomass with CCI before the filling stage during 2016–2017.

## Conclusions

In this study, we introduced a biochemical photosynthetic component, leaf chlorophyll, as a physiological calibration parameter to estimate AGB in crop simulations. Good simulation results were obtained with leaf CCI, which demonstrated the significance of using leaf chlorophyll for modelling AGB. The simulations provide crop growth details, which can help in our understanding of biomass dynamics, accumulation and crop growth within a field. We also compared the dynamics of LAI, CCI, and PAR_*i*_ with the shifts in phenology during 2016–2017. These comparative results were an effective way to improve model performance by reducing the errors in the observed values, especially before the maturity stages of rice. Our statistical diagnostic results also confirmed that the adjustment approach with CCI increased the precision of the AGB simulations. The proposed rice estimation method for AGB that was based on leaf CCI, PAR_*i*_, and LAI is a promising way to estimate rice AGB using computer simulation technology. This study provided an approach that can enhance estimation of AGB in the future.

## Supplemental Information

10.7717/peerj.6240/supp-1 Supplemental Information 1 Raw data.

10.7717/peerj.6240/supp-2 Supplemental Information 2 Comparison of model 1, 2 and chlorophyll concentration.

## Additional Information and Declarations

### Data Availability

The following information was supplied regarding data availability: Raw data is provided in the Supplemental Files.
